# Wooden owl that redefines Earth's biosphere may yet catapult a fungus into space

**DOI:** 10.1111/1462-2920.14510

**Published:** 2019-01-24

**Authors:** John E. Hallsworth

**Affiliations:** ^1^ Institute for Global Food Security, School of Biological Sciences, Queen's University Belfast, MBC 97 Lisburn Road, Belfast BT9 7BL UK

Cellular systems are vulnerable to environmental parameters that impose thermodynamic constraints on metabolic function. These include biophysical challenges and events relating to low water‐availability, solute‐induced stresses, dehydration‐rehydration cycles and extremes of temperature, and these can sometimes be lethal. The concept of a water‐availability limit for Earth's biosphere might evoke the Sahara, Atacama and other deserts where water is scarce, or subzero environments where any water is locked up as ice. However, if a cell is dried then, whereas it may remain viable, life processes cease, from an existential viewpoint it is debatable whether life exists/occurs under such conditions at all. Regardless of this, water stress also occurs in microbial habitats that are wet, even within bodies of water. Indeed, it is arguably implausible that any microbial system can ever function in a stress‐free mode, and this can often be due oxidative stress caused by reactive oxygen species as well as oscillating suboptimal or supraoptimal water activity (Hallsworth, 2018).

**Figure 1:**
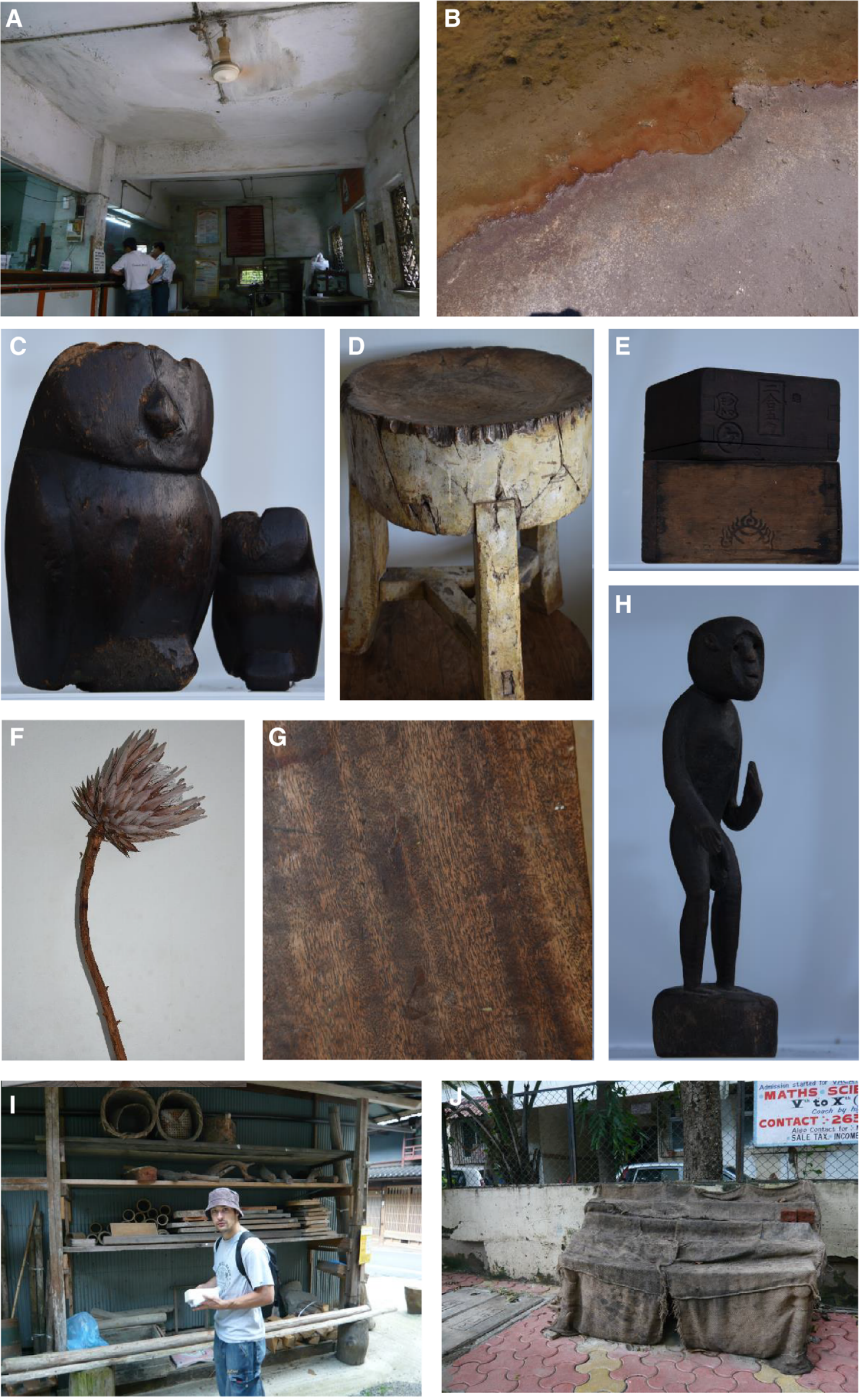
Habitats of xerophiles and halophiles: (A) melanised fungal biomass visible on surfaces within a building (Vile Parle West, India), (B) a natural crystalliser pond in Alviso, (San Francisco Bay, CA, USA), such as those where the most‐extreme halophiles have been found (Javor, 1984; Antón *et al*., 2000; Stevenson *et al*., 2015b), (C) wooden owls; apparently old, and made in Thailand (purchased in Paris, France, 2004); (D) > 100‐year‐old sycamore chopping‐block (purchased in Derbyshire, UK, 2006), (E) > 100‐year‐old wooden rice measure (*masu*), made in Japan (purchased in Kumamoto, Japan, 1997), (F) stem of a dried *Protea* flower (in UK, 2006), (G) approximately 150‐year‐old mahogany table top (purchased in Carlisle, England, UK in 1990); (H) antique wooden statue, made in India (purchased in Kumamoto, Japan, 1997), (I) Jim P. Williams about to sample the surfaces of dried bamboo (Kurama, Japan in 2006) and (J) jute rug in India (Mumbai, 2008). For details of isolates obtained from (C to I), see Williams and Hallsworth (2009). NB: Whereas the author purchased items C–H, their provenance prior to this cannot be confirmed.

Water activity, that is the mole fraction of water molecules in a solution, is a potent stress parameter that is numerically equivalent to relative humidity but divided by 100 (Scott, 1957). Cells respond to low water activity, osmotic stress and other water‐ and solute‐induced stresses using a variety of mechanisms, including production of protein‐stabilization proteins, increased generation of cell‐available energy, adaptations of plasma‐membrane composition and accumulation of soluble stress‐metabolites (compatible solutes) that can act in osmotic adjustment and protect macromolecular structures, depending on the microbe and stress(es) experienced. Some microbes, known as xerophiles, are able to function optimally at much lower values of water activity than most other taxa. Here, ‘xerophiles’ refers to fungi that are capable of growth at low water activity on high‐sugar or high‐glycerol media (although some xerophilic fungi are also halophilic). Research into this group was historically driven by the food industry, with studies focusing on fungi that spoil high‐sugar foods (Gane, 1950; Scott, 1957; Pitt, 1975; Dagnas and Membré, 2013; Biango‐Daniels and Hodge, 2018). In recent years, an increasing amount of information has come to light about fungi in high‐salt habitats, such as solar salterns (Gunde‐Cimerman and Zalar, 2014; Nazareth and Gonsalves, 2014). Although some xerophiles are known to cause indoor contamination of building materials (Fig. 1A) and the formation of brown spots on paper (caused by the Maillard reaction and known as ‘foxing’: Arai, 2000; Piñar *et al*., 2015), the surfaces of domestic furniture and artefacts have generally been overlooked as a potential source of extremely xerotolerant microbes.

Water availability is a potent determinant for life on Earth, and it is a quirk of history that water activity was designated a maximum value of 1. The parameter is derived using Raoult's Law (Scott, 1957), so we designate water activity in decimal fractions. For 50 years, the water‐activity value at which the most‐resilient microbe was thought to become non‐functional is 0.605 (Pitt and Christian, 1968); equivalent to a relative humidity of 60.5%. In other words, whereas the biophysical window for microbial life on Earth spans a temperature range of approximately 160°C, the entire water‐activity window for life is allocated a value of only 0.4 units (Stevenson *et al*., 2015a). Given that living systems are as sensitive to water stress as they are to temperature, this makes it is easy to overlook the profound impact of water activity on cellular systems.

Optimal rates of growth/metabolism, and/or health, occur at different water‐activity values, according mainly to the biological system. In humans and most other mammals, this is approximately 0.995 (Pirt and Thackery, 1964; Persons *et al.,* 1987), for most fungi approximately 0.990 and for some extreme xerophiles 0.900–810 (Stevenson *et al*., 2015b; 2017a). In their study, Pitt and Christian isolated fungi from spoiled fruits, and determined the water‐activity minima at which they were able to germinate. *Aspergillus*, *Chrysosporium*, *Penicillium* and other genera were present, but the most xerophilic was a *Xeromyces bisporus* strain (FRR 0025) that germinated at 0.605 water activity after a four‐month incubation. For organismal systems, cellular water‐activity tends to be closely regulated, although some plant systems and some invertebrates are able to undergo partial desiccation via anhydrobosis. Many fungi can *survive* dehydration but for mammals, such as ourselves, relatively small fluctuations of cellular water‐activity are lethal. In relation to *X. bisporus*, 0.605 has been used as the seminal value upon which methodologies to preserve foods, museum specimens, artworks, documents and books have been based. In addition, the COSPAR Planetary Protection Policy, recognized by the United Nations, is based this value. This policy minimizes the risk of contamination events on extraterrestrial bodies with terrestrial microbes during space‐exploration missions (Kminek *et al*., 2010; Rummel *et al*., 2014). Whereas *X. bisporus* has held its position as the most‐extreme xerophile for five decades, there was little systematic research carried out during this time to find microbes that might be more xerophilic. It seemed difficult, therefore, to accept that strain FRR 0025, which originated from a prune, might not one day be dislodged from its number‐one position.

So, I set about trying to coax various microbes to multiply below this value. Optimistically, and with some naivety, we expected that achieving this would take several months. It transpired, however, that this scientific journey which began in 2001 threaded its way through several generations of research students and the three domains of microbial life; involved 126 collaborators/coauthors (from 22 countries), and sampling campaigns on two different continents; and spanned a period of almost 20 years.

Working as a PhD student with Naresh Magan (Cranfield University, England, UK; 1991–1994), we exploited the phenotypic plasticity of entomopathogenic fungi to enhance intracellular accumulation of the compatible solutes trehalose, mannitol, arabitol, erythritol and glycerol. We found that the lowest molecular‐weight polyols, especially glycerol, increased the vigour of, and reduced the water‐activity minimum for, fungal germination and growth *in vitro*, and on the insect host (Hallsworth and Magan, 1994; 1995). After this, I took several postdoctoral positions, at Heriot‐Watt University (Scotland, UK), Kumamoto Institute of Technology (Japan), Stellenbosch University (South Africa) and then – in the group of Kenneth N. Timmis – at University of Essex (England). The microbial model system(s) and their applications differed in each location, but the underlying theme was consistent; to identify cellular stress mechanisms and responses. This included stresses imposed by low‐temperature and low water‐activity, and that imposed by ethanol, urea and other chaotropic substances; that is those that entropically disordering macromolecular systems (i.e. those that are chaotropic). Whereas chaotropicity and water activity are mechanistically distinct stress parameters, the chaotrope‐stress work impacted our search for microbes that can tolerate low water‐activity.

During the time in Essex, I worked on several projects, including the EU‐funded Biotechnologies from the Deep (BIODEEP; 2001–2004), alongside halophile expert Terry J. McGenity. BIODEEP focused on the microbial ecology of the Mediterranean deep‐sea anoxic brine lake *Discovery* that lies 3.58 km below the ocean surface. The brine of the lake body is saturated with MgCl_2_, and the lake's interface with the overlying seawater is a 1.5‐m deep halocline with a Mg^2+^ gradient from 50 mM to 5.05 M (Hallsworth *et al*., 2007). Our study was carried out in collaboration with Michail M. Yakimov (Institute for Coastal Marine Environment, Italy), Peter N. Golyshin (Helmholtz Centre for Infection Research, Germany) and others, and the main scientific finding was that the chaotropicity of MgCl_2_ constrained life to the upper layers of the interface. There was no metabolic activity in evidence in the lower interface at MgCl_2_ concentrations of > 2.3 M. Furthermore, disparate lines of evidence indicated that the presence of kosmotropic (macromolecule‐stabilizing) salts would enable microbial activity at slightly higher MgCl_2_ concentrations, by mitigating against the chaotropicity of the latter; a phenomenon that was confirmed in a number of other studies (Williams and Hallsworth, 2009; de Lima Alves *et al*., 2015; Cray *et al*., 2015; Yakimov *et al*., 2015).

We also worked on NaCl‐saturated deep‐sea systems (Daffonchio *et al*., 2006), and noticed that some halophilic bacteria and Archaea exhibit very high growth rates – and even optimal growth – at NaCl saturation (0.755 water activity). However, proliferation has not been observed at lower water activity due to the finite solubility of NaCl. During the Lake *Discovery* study, we also worked on synthetic, MgCl_2_‐dominated substrates and found that no halophiles can retain function in such brines even at 0.755 water activity (Hallsworth *et al*., 2007). So, we set about looking into how we might manufacture brines with a water‐activity of < 0.755, yet a sufficiently low chaotropic activity to permit halophile metabolism. At the time, one of our colleagues commented that this kind of work ‘seems a bit like breaking records’. This was an amiable colleague, and the comment was meant to be helpful, but we didn't share that view. For me, working with life at the point where the cellular system interfaces with extreme, thermodynamic constraints is in itself motivating, and even fascinating. Understanding system failure at the biophysical limit can potentially elucidate aspects of ecophysiology even for non‐extremophilic microbes. We also believed that water plays a key role under the extreme (solute‐induced) conditions where even the most‐resilient microbes fail (Hallsworth *et al*., 2003; Stevenson *et al*., 2015a; Ball, 2017).

We recruited MSc student Richa Sahay (2003–2004) for the study of halophiles at low water‐activity. Richa cultured some of the most halophilic microbes known, and determined ability to grow in synthetic brines that contained NaCl+MgCl_2_ and NaCl+MgCl_2_ + either glycerol or ethylene glycol (and some nutrients). In this way, we found that some strains can indeed proliferate below 0.755 water activity, and even down to 0.681. Analyses of data from published sources (e.g. Javor, 1984; Yoshida *et al*., 1991; Antón *et al*., 2000; Bolhuis *et al*., 2004; Deole *et al*., 2013) were later carried out by PhD student Andrew Stevenson from 2013 to 2016, who worked with me once based in Belfast. To ensure that our study was as comprehensive as possible, we asked a number of experts to join us: Ailsa D. Hocking (fungal xerophiles; CSIRO Food and Nutrition, Australia), Nina Gunde‐Cimerman (fungal halophiles; University of Ljubljana, Slovenia) Josefa Antón and Aharon Oren (halophilic bacteria and Archaea; University of Alicante, Spain and The Hebrew University of Jerusalem, Israel respectively) and others. Andy's analyses revealed that some prokaryotes function at even lower water‐activity values; that is down to 0.635 (Fig. 1B). Collectively, the findings showed that the respective limits for the three domains‐of‐life actually converge close to a common water‐activity value (Stevenson *et al*., 2015b). Additional studies, carried out by undergraduate student Callum J. D. Lee *et al*., confirmed that NaCl‐saturated environments are moderate rather than thermodynamically extreme habitats for microbes; that is given that microbial ecosystems can function at < 0.755, saturated NaCl does not represent a water‐activity barrier to the microbial biosphere (Lee *et al*., 2018).

Jonathan A. Cray (PhD student from 2012 to 2015) became involved in our water‐activity studies through his analyses of deep‐sea brines (Yakimov *et al*., 2015). The scientific journey also wove its way through the stress biology of pathogenic aspergilli (Paulussen *et al*., 2017), and the biophysics of high‐sugar ecosystems (Lievens *et al*., 2015). We also examined the evidence for ecosystem function, based on the functionality of the most‐extreme xerophiles and halophiles at < 0.690 water activity (Stevenson *et al*., 2015a). Whereas there is an active international research community working on halophiles (this term gives 449 hits on the Web‐of‐Science database for the past five years; at 5 November 2018), fungal xerophiles are less‐extensively studied (the term ‘xerophile*’ yields only 22 hits). The relatively low level of interest in xerophiles however, seems paradoxical given the important applications of fungal xerophiles, and the insights they offer into biophysics of life.

Some of our other studies at the time of Richa's halophile study were focused on fungi and a Brazilian postdoc., Flávia de Lima Alves, joined us at University of Essex to work on this topic (2003–2004). Flávia's studies of solute‐stress tolerance in *Aspergillus wentii* indicated that glycerol was the most biologically permissive solute that can be used to reduce the water activity of nutrient media (de Lima Alves *et al*., 2015). They also revealed that glycerol induces an osmotic change only momentarily, and then quickly equilibrates across the plasma membrane. At the same time as Richa and Flávia were working on solute‐induced stresses, I made a recreational trip to Paris, visited a street market there, and purchased two wooden (mother‐and‐baby) owls that, according to the vendor, had been made in Thailand (Fig. 1C). This trip to the Parisian market, it turned out later, impacted our research trajectory.

I began setting up a research group in Queen's University Belfast (Northern Ireland, UK) from 2006, and recruited PhD student Jim P. Williams at this time (2006–2009). He is an experiment‐driven scientist with deep interest in cellular biology, and his sponsor provided blue‐skies funding for the project. So, we were able to focus on the (arguably esoteric) search for microbial proliferation below 0.605 water‐activity. We obtained xerophile strains by scouring the literature for extreme xerophiles and obtaining these from commercial culture collections, and via sampling campaigns in three humid climates: cool Northern Ireland and relatively hot regions of India and Japan. We began sampling activities by swabbing the surfaces of wooden structures located outdoors, and surfaces of furniture and domestic artefacts; some of these in my own home. The collection we amassed, 157 isolates in total, also included the *X. bisporus* strain (FRR 0025; i.e. ATCC 28298) that was isolated by John I. Pitt and used in the study by Pitt and Christian (1968). For all xerophile isolates, Jim began by assessing ability to grow at low water‐activity using ethanol, NaCl, urea, ethylene glycol, KCl, ammonium nitrate, glycerol, MgCl_2_, guanidine hydrochloride, CaCl_2_, glucose and sorbitol as stressors, and established that the majority of strains grew at their lowest water‐activity on glycerol‐supplemented media.

Glycerol, a small and polar molecule with behaviour that is not dissimilar to that of water, appeared to play essential biophysical and ecophysiological functions in many microbial systems under extremely hostile conditions, so we developed an intense interest in this polyol. This, in turn, gave rise to a thematic issue of *Environmental Microbiology* (‘Environmental Glycerol Metabolism’; Hallsworth, 2017) and a presentation at the International Symposium on Fungal Stress (in Brazil, 2017; see Alder‐Rangel *et al*., 2018) ‘A Story about Glycerol’. The latter focused on the main characteristics of glycerol as both cellular stress‐protectant and cellular stressor; this polyol:

- accumulates in conidia produced at low water‐activity, enhances germination of conidia on solid media at low water‐activity and facilitates host infection at low relative humidity by entomopathogenic fungi (see above),
- is chaotropic at high concentrations (Williams and Hallsworth, 2009; Cray *et al*., 2013),
- reduces water activity more effectively than other kinds of organic compatible solute (de Lima Alves *et al*., 2015),
- can act hydraulically to facilitate infection of the plant host by fungal plant pathogens (Foster *et al*., 2017),
- can (paradoxically) mitigate against stresses induced by chaotropes and chaotropicity‐mediated hydrophobe‐induced stress (Hallsworth *et al*., 2003; Bhaganna *et al*., 2010; 2016),
- is the most permissive solute‐stressor for growth of xerophiles at low water‐activity (Williams and Hallsworth, 2009),
- inhibits growth and metabolism via the reduction of water activity and, at high concentrations, via chaotropicity (but is not an osmotic stressor) (de Lima Alves *et al*., 2015) and
- can enhance cold tolerance and reduce the low‐temperature minima for growth by facilitating flexibility of cellular macromolecules (Chin *et al*., 2010; C. L. Magill and J. E. Hallsworth, unpubl. data).

Jim assayed xerophile growth rates over a matrix of conditions (water activity, temperature, pH) and selected the 25 most‐xerophilic strains, each capable of growth at ≤ 0.750 water activity. These included *Aspergillus penicillioides* JH06THH and JH06THJ (isolated from the mother owl, Fig. 1C), JH06GBM and JH06GBO (from the underside of an antique sycamore chopping‐block from the UK, Fig. 1D), FRR 2179 (from dried chillies), *E. repens* JH06JPD (from an antique wooden rice‐measure purchased in Japan, Fig. 1E), *Eurotium amstelodami* FRR 2792 (from a date fruit), *Xeromyces bisporus* FRR 0025 (from a high‐moisture prune), FRR 3443 (from 0.656‐water‐activity raisins) and FRR 2347 (from a 0.750‐water‐activity, mouldy fruit cake) (Williams and Hallsworth, 2009). Those strains isolated during our own sampling campaigns (including JH06THH, JH06GBM, JH06GBO and JH06JPD) were identified using a combination of classical and sequencing approaches (Williams, 2010). *Aspergillus penicillioides* strains JH06THH, JH06GBM and JH06GBO, *Eurotium repens* JH06JPD and strains JW07GB158, JH06IN47 and JH06IN48 (see below) were isolated from wooden objects (Fig. 1C–H) that had been kept in the author's 1922 house, which is located close to the Northern Ireland coast, built of clay brick and wood, partly on bedrock and clay subsoil with a slate roof tiles, wooden floorboards, fired‐clay floor tiles (and without double glazing or modern insulation). It may be that many such buildings provide favourable habitats for subsisting xerophile communities. In order to establish the water‐activity minimum for mycelial growth, Jim inoculated selected xerophiles onto a range of nutrient media supplemented with glycerol or glycerol plus kosmotropic solutes (e.g. NaCl, KCl, sucrose). These studies identified some strains capable of hyphal growth at lower water‐activity values that had been reported previously (i.e. in the range 0.640–0.653) (Williams and Hallsworth, 2009; Stevenson *et al*., 2015b). These were: at 0.640 water activity, *A. penicillioides* JH06THJ and *X. bisporus* strains FRR 2347 and FRR 3443 (Stevenson *et al*., 2015a); at 0.647, *A. penicillioides* strains JH06GBM, JH06GBO, JH06THH and FRR 2179; and at 0.653, *X. bisporus* strains FRR 1522 (Williams and Hallsworth, 2009). We were fascinated by the finding that chao‐/kosmotropicity impacted water‐activity minima for xerophile function, but at the same time disappointed not to have observed biotic activity at ≤ 0.605.

The 1968 finding that *X. bisporus* can germinate at 0.605 had not been equalled or surpassed during subsequent studies of fungal xerophiles. Furthermore, whereas extrapolations of growth curves suggest a theoretical minimum for some halophiles of down to 0.611, there are no empirical determinations of biotic activity of halophilic bacteria or Archaea at ≤ 0.635 (Stevenson *et al*., 2015b). Occasional studies have reported high levels of microbial metabolism and growth at exceptionally low water‐activity values: growth of mesophilic actinomycetes at 0.500 (Doroshenko *et al*., 2005; Zvyagintsev *et al*., 2012), germination of the fungal xerophile *Wallemia sebi* at 0.600 (Frank and Hess, 1941) and geochemical data in the deep‐sea brine lake *Kryos* at 0.400 and purported to be evidence of viable and active sulfate reducers such as *Desulfovermiculus* and *Desulfobacula* (Steinle *et al*., 2018). Such data have not been independently validated, and have either been subsequently disproved (Pitt and Christian, 1968; Stevenson and Hallsworth, 2014) or lack data showing any cellular viability or activity (Steinle *et al*., 2018; M. M. Yakimov *et al*., in preparation).

We carried out analyses of growth kinetics for *X. bisporus* and *A. penicillioides* strains (those with the most‐extremely xerotolerant mycelia), and found that the fungal cell is sensitive to changes of water activity of at least ±0.001 (Stevenson *et al*., 2015a). We therefore fine‐tuned the methodology for water‐activity determination, based on Novasina humidity‐sensor technology (Novasina AG, Lachen, Switzerland), to be reproducible to an equivalent level of accuracy (Hallsworth and Nomura, 1999; Stevenson *et al*., 2015b).

Fungi growing on a wooden surface might obtain water from both wood and atmosphere, and may also generate water via cellular metabolism. Assuming that the mole fraction of water in wood and the vapour phase are in disequilibrium (i.e. water activity and relative humidity are not equivalent), the fungal cell would most‐easily access water from the phase with the highest water‐activity/relative‐humidity value, but little work has been done on this topic. From 2103 to 2014, the Mars Exploration Program Analysis Group (MEPAG) ‘Special Regions’ Scientific Analysis Group (SR‐SAG)2 (of which I was a member) was taking place (Rummel *et al*., 2014), and we considered whether microbes could proliferate by absorbing water from the vapour phase alone. The idea that microbes may be able to grow without any exogenous source of liquid water was a controversial topic, giving rise to some debate on SR‐SAG2 teleconference calls, and at our face‐to‐face meetings. One intriguing issue, raised by SR‐SAG2 member Thomas L. Keift (New Mexico Institute of Mining and Technology, NM, USA), was the possibility of thin aqueous films that can form on surfaces spontaneously. So, microbes growing on surfaces not known to receive condensation may potentially receive a supply of liquid water via thin‐film formation that is invisible to the naked eye. This inspired me to begin a collaborating with Jürgen Burkhardt (University of Bonn, Germany), a physicist carrying out pioneering studies into thin films on plant surfaces. We used a movie of thin‐film formation on a plant surface to explain how thin films can act as sources of unseen liquid water for microorganisms (Stevenson *et al*., 2015a). Microbial cells are known to absorb water from the vapour phase, and to generate water through metabolic activities, but whether or not an external source of liquid water is required for microbial proliferation remains unresolved.

Andy Stevenson was in the final six months of his PhD work at Queen's when we decided to have a final attempt to re‐examine the water‐activity limit for fungi, via studies of germination on a range of media supplement with glycerol or glycerol plus other solutes. A number of xerophiles were selected for this study: *X. bisporus* strains FRR 0025, FRR 1522 (from spoiled licorice), FRR 2347 and FRR 3443, *Eurotium amstelodami* strain FRR 2792, *Eurotium echinulatum* strain FRR 5040 (from sultanas), *Eurotium halophilicum* strain FRR 2471 (from soil), *Xerochrysium xerophilum* (formerly *Chrysosporium xerophilum*) strain FRR 0530 (from spoiled high‐moisture prunes; from the CSIRO Food and Nutritional Sciences Culture Collection, North Ryde, Australia) and *A. penicillioides* strains JH06GBM, JH06THH and JH06THJ and *E. repens* strain JH06JPD (Stevenson *et al*., 2017b). Fungal spores that contained high levels of intracellular glycerol were obtained from cultures grown on glycerol‐supplemented media. They were harvested in solutions of glycerol, or glycerol plus other solutes and these spore suspensions were used to inoculate the range of low water‐activity media. As expected, this high‐glycerol study system gave rise to unprecedented levels of germination vigour at low water‐activity, and reduced the water‐activity minima for germination of individual strains. Counter to our expectations, however, germination did not occur at ≤ 0.605; *X. bisporus* germinated down to 0.637, *A. penicillioides* to 0.640 and *Eurotium* (*E. halophilicum*) to 0.651 water activity. So, once again our attempts to observe microbial activity at ≤ 0.605 had been frustrated. Yet, intriguingly, extrapolations indicated a theoretical water‐activity minimum for germination of *X. bisporus* and *A. penicillioides* of 0.570. Several colleagues explained to us that extrapolations are theoretical and that there may be no biotic activity in reality below 0.605. I accepted the rational beneath such comments, but also felt that these extreme xerophiles were capable of more than we had observed within our experimental system.

**Figure 2:**
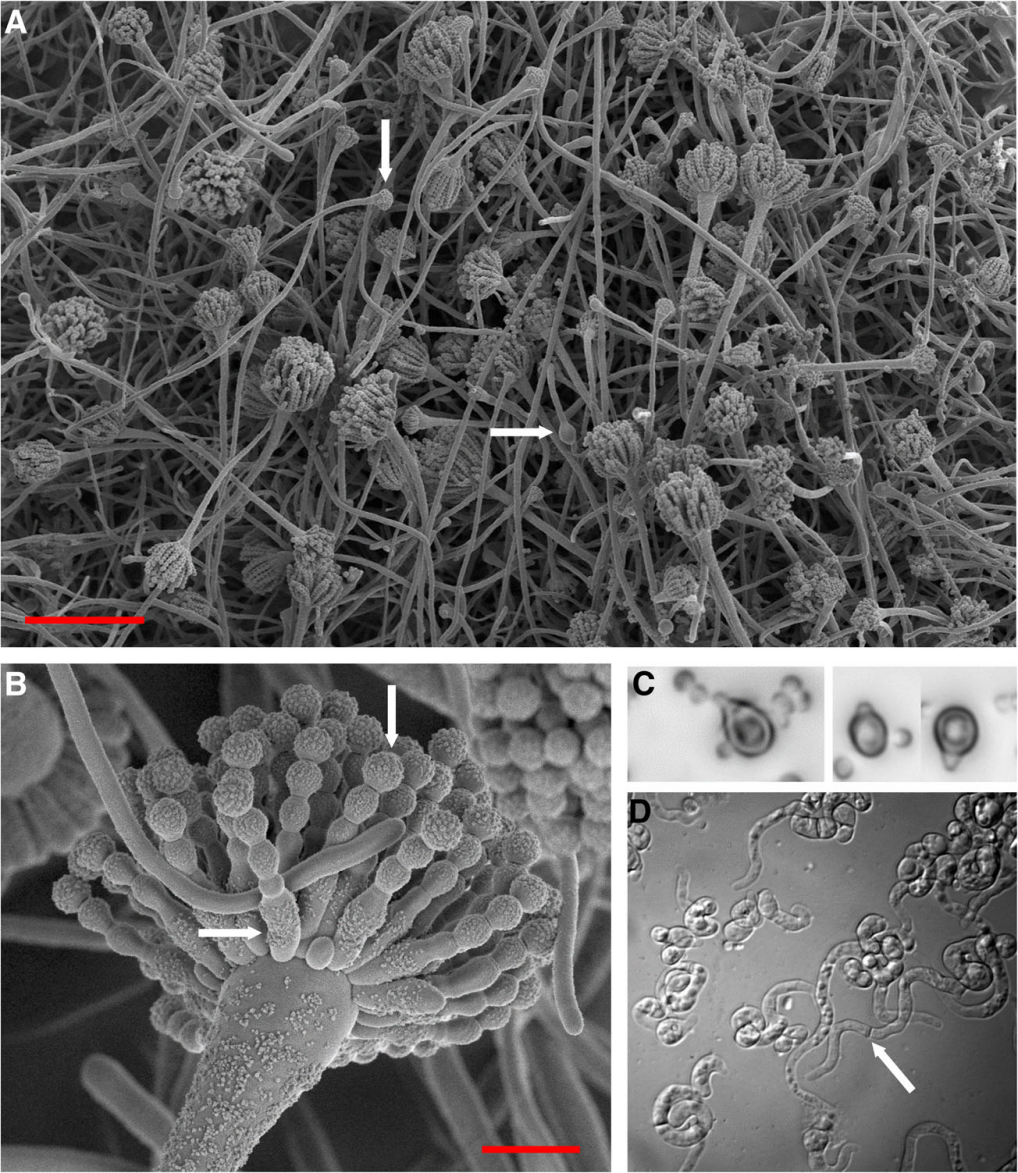
*Aspergillus penicilliodes* (JH06THJ) showing (A) sporulating mycelium and (B) a conidiophore from a culture growing on malt extract agar supplemented with 5 M glycerol (0.844 water activity; after 47 days at 24°C); (C) differentiated germination structures which have a polarized, tapered morphology at 0.598 water activity (18 days) on malt extract yeast extract phosphate agar supplemented with glycerol + NaCl + sucrose; and (D) septate germlings at 0.585 water activity (57 days) on malt extract yeast extract phosphate agar supplemented with glycerol. Images (A) and (B) were produced by Jan Dijksterhuis using scanning electron microscopy and the red scale bars indicate 100 and 10 μm respectively. Images (C) and (D) were taken using a Nikon MULTIZOOM AZ100 microscope (Χ400) and are modified from Stevenson *et al*. (2017c), where further methodological details can be found. Arrows indicate: (A) early‐stage conidiophore formation (horizontal arrow) and mid‐stage conidiophore formation (vertical arrow); (B) a phialide (horizontal arrow) and an ornamented conidium (vertical arrow); (C) a septum of a germ tube.

In the final weeks of his PhD project by this time, I pleaded with Andy to try one final experiment. Many xerotolerant species were obtained from our sampling campaigns, including *Eurotium niveoglaucum* JH06GBb from the stem of a dried *Protea* flower in UK (2006; Fig. 1F); *Aspergillus vitricola* JW07GB158 from an antique mahogany table top, UK (2006, Fig. 1G); *Penicillium glabrum* JH06IN47 and JH06IN48 from a wooden Indian sculpture (purchased in Japan, 1997; Fig. 1H), *A. vitricola* JW07JP117a from the internal surface of a dried section of bamboo in Japan (2006; Fig. 1I) and *Aspergillus niger* JH08IN49 and JH08IN51 from jute fabric (Fig. 1J) in India (Williams and Hallsworth, 2009; Williams, 2010). The most xerophilic isolates, however, came from the surfaces of wooden objects (Fig. 1C–E) (Williams and Hallsworth, 2009). Initial studies that were carried out to optimize assay parameters such as temperature and pH established that *A. penicillioides* strain (JH06THJ) may be the most xerophilic strain in our collection (Fig. 2A and B); it had both germinated and exhibited hyphal growth at 0.640 (Stevenson *et al*., 2015b; 2017a,b). Furthermore, *A. penicillioides* has an intriguing and idiosyncratic ecology, and is a generalist that is environmentally ubiquitous (Stevenson *et al*., 2017c; W. K. O'Neill and J. E. Hallsworth, in preparation) despite being marginally more xerophilic than *X. bisporus* that is well known for being an extreme specialist (Leong *et al*., 2015). So, for our final experiment, we focused on JH06THJ, the isolate obtained by swabbing the owls from the Parisian street market (Fig. 1C).

We modified the germination assay slightly to avoid water loss from the culture system, and spores were dry harvested prior to inoculation to avoid any intermediate steps in the procedure. Also, rather than assessing + or – germination, a multidimensional picture of the process was obtained by quantifying: rates and extent of conidial swelling during the uptake of water, production of differentiated germination structures, formation of septate germlings, and development of mycelium and/or sporulation: elaboration of branched, adventitious germ‐tubes followed by production of aerial hyphae and/or production of conidiophores and conidia. For this purpose, a range of 18 test media were used, giving a water‐activity range from 0.741 to 0.585 (Stevenson *et al*., 2017c). The germination kinetics in our previous study indicated that JH06THJ should be capable of germination on most of these media, even the three at < 0.605 water activity (Stevenson *et al*., 2017b). Whereas there were lower water‐activity media, extrapolations of germination curves indicated that these stressor combinations would not permit germination at their respective water‐activity values. We decided to work with the mycologist Jan Dijksterhuis (Westerdijk Fungal Biodiversity Institute, The Netherlands) who has extensive expertise as a microscopist. So Andy travelled to the Westerdijk Fungal Biodiversity Institute with the conidia of strain JH06THJ already inoculated onto his low water‐activity culture media. The week‐long visit to Jan's laboratory had been timed with the hope of catching the early stages of germination but, as the week progressed, it became apparent that the process had not yet begun. Jan therefore monitored the plates after Andy's return to Belfast. Gradually, the owl isolate did begin to germinate; first on glycerol+sucrose nutrient media at 0.734, then on some other media, and then by – day 11 – there was differentiation apparent at 0.585 (a glycerol‐supplemented medium); that is production of polarized, tapered germination structures. By day 57, cell division had taken place at 0.585; fully formed septate germlings had been produced (Fig. 2C and D). Strain JH06THJ responded well to manipulations of chao‐/kosmotropicity, but the 0.585 water‐activity germination had occurred on a medium supplemented with glycerol alone. Regardless, after so many years of effort, we had finally observed differentiation and cell division of a microbe at < 0.605 water activity. As explained in Stevenson *et al*. (2017c), this finding represents a 5% increase in the water‐activity window for microbial life, and this is to an 8°C expansion of the temperature window for life. It seems paradoxical that the most xerophilic activity was observed on a high‐glycerol (chaotropic) medium, although it may be that trehalose within the conidia mitigated this chaotropicity. In fact, extrapolations of data indicated an even lower theoretical water‐activity minimum; of 0.565.

When we wrote up the previous multi‐xerophile study in which the high‐glycerol experimental system was found to be pivotal (‘Glycerol enhances fungal germination at the water‐activity limit for life’; Stevenson *et al*., 2017b), we focused on the biophysical role of glycerol. Therefore, when writing up the 0.585 water‐activity study, we instead explained the finding from an astrobiological perspective. The finding has since generated some interest from the document‐preservation and astrobiology sectors, including invitations to the COSPAR panel for the 2nd Workshop on Refining the Planetary Protection Requirements for Human Extraterrestrial Missions in 2018 (Lunar and Planetary Science Institute, Houston, TX, USA), and to join the Lichens and Fungi Experiment (LIFE) and the Icy Exposure of Microorganisms (ICE_x_POSE) research teams. The LIFE study exposed black fungi to space conditions on the outside of the International Space Station during a 1.5‐year study (Onofri *et al*., 2012; 2018). The ICE_x_POSE project has been granted European Space Agency permission to use the Concordia research facility to expose *A. penicillioides* JH06THJ and other microbes to space‐like conditions during a long‐term experiment in the Antarctic. This facility is 3233 m above sea level on the Antarctic Plateau and thought to be the coldest, driest place on Earth, with an annual mean air temperature of −54.5°C and summer temperatures rarely rising above −25°C (Timmery *et al*., 2011; Michaud *et al*., 2014). Ultimately, this project may lead on to a study of space exposure for strain JH06THJ on the International Space Station. So, the search for an extreme xerophile was an unexpectedly long and perplexing one. Shopping in Paris led to the wooden owls; these provided an extremely stress‐tolerant *Aspergillus*; this fungal strain led to a series of new collaborations and friendships, and a number of astrobiology‐focused activities.

On the owl surface, *A. penicillioides* JH06THJ was presumably at values of water activity or relative humidity greater than 0.585 or 58.5% because the humidity within a NI home can often exceed this value. However, such xerophiles can survive long periods of inactivity when dehydrated, and there may have been times of inactivity whenever water was less available. Brown (1976) once tabulated a ranking of microbial taxa on the basis of their water‐activity minima that had been compiled by J. I. Pitt (CSIRO Food and Nutrition), which to an extent focused on food‐spoilage microbes, and has been modified and re‐used by others many times since (e.g. Grant, 2004). On a recent trip to India, I was admiring the microflora of a mango‐tree branch because, whereas the microbial community was biodiverse, there was clear zoning of taxa based on position on the branch that was apparently determined by water availability. To me, this tree presented a living version of Brown's classic table, with the microbial biosphere represented in technicolour – and in relation to water activity – across a series of environmental gradients. No doubt, gradients of water activity also occur on mm‐, to nm‐scales (or less), such as where water is evaporating from soil particles or other surfaces, including those of the human body. The water‐activity boundary for life is, in terms of thermodynamics, a concrete phenomenon, yet one that can be both dynamic and ephemeral. For example, the water of some microbial habitats that contain sugar (e.g. nectar) (Witt *et al*., 2013; Lievens *et al*., 2015) can evaporate causing rapid and profound changes in water activity. The same phenomenon occurs in sea spray, on rock surfaces and hypersaline brine systems (Benison *et al*., 2007; Michaud *et al*., 2018). Similarly, microbes within bioaerosols may experience being in pure water (water activity = 1) and as the droplet evaporates, the cell may be without liquid water (Mao *et al*., 2007; Verreault *et al*., 2008). This illustrates how fraught the notion of a spatial limit for life can be. It also underlines that the edge of Earth's biosphere is all around us and, as the owls also demonstrate sampling in unobtrusive places can yield new microbes and new science.
